# Changes in the use of e-cigarettes to stop smoking among adults following the rise of disposable vapes: a repeat cross-sectional survey 2016–2023 in England

**DOI:** 10.1136/bmjph-2025-004422

**Published:** 2026-06-28

**Authors:** Loren Kock, Sarah Jackson, Lion Shahab, Harry Tattan-Birch, Hazel Squires, Jamie Brown

**Affiliations:** 1 Department of Behavioural Science and Health, University College London, London, UK; 2 Sheffield Centre for Health and Related Research, The University of Sheffield, Sheffield, UK

**Keywords:** Public Health, Epidemiology, Drug Monitoring

## Abstract

**Introduction:**

Using a representative repeat cross-sectional survey, we investigate how the prevalence of e-cigarette (‘vape’) use, prescription or specialist support and other (non-prescription or specialist support) methods in smoking quit attempts changed following growth in novel disposable vape use in England.

**Methods:**

8323 adults (≥18 years; 47.5% women; mean (SD) age: 39.2 (15.5)) who smoked—and tried to quit—in the past year, were surveyed between July 2016 and December 2023. Using segmented regressions, we estimated annual trends in smoking quit attempts using (1) a vape, (2) prescription or specialist support and (3) other methods before (‘pre-disposables’) and after June 2021 (‘post-disposables’). In a sensitivity analysis, we modelled changes in each outcome associated with the prevalence of disposable vaping as a continuous variable.

**Results:**

During the pre-disposables period, vape use in a quit attempt decreased by 3.1% per year (relative risk (RR)=0.969 (95% CI 0.927 to 1.012)). This trend reversed when disposables became popular (RR=1.20 (95% CI 1.12 to 1.29)), with a relative year-on-year increase in prevalence of 16.5% from June 2021 (from 27.7% in June 2021 to 40.6% in December 2023). Conversely, use of other (non-prescription or specialist support) methods increased by 3.6% per year (RR=1.036 (95% CI 1.01 to 1.06)) before June 2021, before reversing (RR=0.91 (95% CI 0.88 to 0.95)) into a relative yearly decline in prevalence of 5.6% (from 65.4% to 56.6%). Use of prescription/specialist support declined by 12% per year (RR=0.880 (95% CI 0.802 to 0.966)) before June 2021, and there was no apparent change in trend. In aggregate models, for each percentage-point increase in disposable vaping, vape use in a quit attempt (in the subsequent month) rose by ~0.66 percentage points (95% CI 0.05 to 1.26), while use of other methods declined by 0.82 percentage points (95% CI −1.40 to −0.24).

**Conclusions:**

Increasing use of disposable vapes in England was associated with increased vape use and decreased use of other (non-prescription or specialist support) methods in smoking quit attempts.

**WHAT IS ALREADY KNOWN ON THIS TOPIC**

- E-cigarette (‘vape’) use has increased substantially among adults in England since 2021, driven primarily by novel disposable vape products.
- This rise in disposable vape use coincided with an increase in vape use in general in smoking quit attempts, but it is unclear whether this growth displaced other effective cessation aids or other quitting methods.

**WHAT THIS STUDY ADDS**

- Following rapid growth of disposable vapes in England, the declining trend in the use of vapes in smoking quit attempts reversed and increased from 2021 onward.
- Rising disposable vape use was associated with a change in the trajectory of vape use in smoking quit attempts, from a declining trend to increasing use in smoking quit attempts from 2021 onwards.

**HOW THIS STUDY MIGHT AFFECT RESEARCH, PRACTICE OR POLICY**

- While the sale of disposable vapes is banned in England, future research and policy should consider the role that similar ‘disposable-like’ reusable devices play in adult smoking cessation, alongside measures to prevent their use among youth.

## Introduction

 Since 2021, e-cigarette use (‘vaping’) increased substantially among adults in England, with approximately 1 in 10 currently vaping by the end of 2023.^1^ This rise occurred predominantly among people who smoke or have a history of smoking but there was also an increase among those who had never regularly smoked.^2 3^ These changes—most pronounced among youth and young adults—were likely caused by growth in new disposable vapes (‘disposables’). Disposables became the most popular device type used by people who vape in England^1^ due to their wide availability in small and large retailers, relatively low up-front cost, high-strength nicotine–salts-based vape liquid and flavour variety.^4 5^ To try and arrest the rising trend in youth vaping and avoid the environmental harms of disposables, the UK government announced plans to ban the sale of disposable devices from June 2025.^6^

Disposable vapes may be an appealing smoking cessation aid,^7 8^ and growth in their use since 2021 has coincided with an increase in the use of vapes in attempts to quit smoking.^9^ There is high certainty evidence that vapes are similarly effective to the ‘gold standard’ cessation support offered by UK stop smoking services (SSS), which combine prescription pharmacotherapy with specialist behavioural support.^10 11^ Therefore, to inform balanced messaging around the UK disposables ban and future vaping regulation, it is important to establish whether rising disposable use altered the trajectory of vape use in quit attempts. In this context, it is also important to assess whether any change represents a shift away from other effective forms of smoking cessation support (ie, vapes displacing similarly effective cessation aids) or a reduction in the use of other methods including those that are less effective such as trying to quit without any support^10 12^ (which includes unsupported attempts to quit).^11^ Moreover, examining how these changes differ across key sociodemographic and smoking characteristics can provide information on whether disposables are reaching groups who may not otherwise have used a vape to stop smoking.

The Smoking Toolkit Study (STS) is an established national survey in England and has captured key changes and trends over time in the use of specific cessation aids, such as the growth and prominence of vaping and the decline of other (previously popular) aids.^13^ Using data from the STS between July 2016 to December 2023, this study aimed to examine (1) how the prevalence of the use of vapes to stop smoking and use of prescription and specialist support and other methods to stop smoking changed following the entry of novel disposable vapes to the vape market in England in 2021 and (2) whether changes differed according to key sociodemographic characteristics and smoking dependence.

## Materials methods

### Preregistration

The study protocol and analysis plan were preregistered on Open Science Framework (https://osf.io/cgjyv/).

### Design

The STS is a representative monthly cross-sectional survey of adults in England.^14^ The study uses a hybrid of random probability and simple quota sampling to select a new sample of ~1700 adults each month. During the COVID-19 pandemic, no data were collected in March 2020 and data collection from April 2020 onwards changed from face-to-face to telephone interviews. Comparisons of the two data collection modalities indicate good comparability.^15^

### Participants

We used data from participants surveyed between July 2016 and December 2023 (before the roll-out of the UK government ‘swap-to-stop’ e-cigarette scheme^16^ and the first announced plans to ban disposable vapes in early 2024).^17^ We selected those who reported that they had smoked and tried to quit in the past year. Because data were not collected from 16-year-olds and 17-year-olds between April 2020 and December 2021, we restricted our sample to those aged ≥18.

### Measures

#### Interruption

We assumed an interruption in the trends between May and June 2021, as has been used in previous studies.^3^ The time series was divided into two segments:

- **Pre-disposables period:** July 2016 (when detailed vaping data were first collected) to May 2021.
- **Post-disposables period:** June 2021 to December 2023, corresponding to the period of increased disposable vape use.^18^

#### Smoking quit attempt

People who smoked cigarettes or tobacco of some kind in the past year (see online supplemental material for full question wording) were asked how many serious attempts to stop smoking they had made in the last 12 months. Those who indicated at least one quit attempt were classified as having made a quit attempt.

#### Outcomes

##### Use of aids to quit smoking

People who made a past-year smoking quit attempt were asked to indicate what method(s) they used to try stop smoking during their most recent serious quit attempt (see online supplemental material for full question and responses).

Those who indicated they used an electronic cigarette or vaping device were classified as using a vape in their most recent smoking quit attempt. Those who reported using a prescribed nicotine replacement product or medication (bupropion, varenicline or cytisine), or a form of specialist behavioural smoking cessation support (a stop smoking group or one-to-one counselling) were classified as using standard evidence-based support typically available through NHS specialist SSS or primary care—hereafter termed ‘Prescription or specialist support’. All others (ie, those who did not use a vape or any of these prescription or specialist support methods) were classified as attempting to quit using ‘other methods’. The determination is based on published research from the STS on the real-world effectiveness of aids to quit smoking in England, and randomised controlled trial (RCT) data, which indicate greater effectiveness of front-line cessation methods (prescription medication and behavioural support).^10 11^ Those classified as using ‘other methods’ include those for which evidence on effectiveness is limited, or where evidence indicates that the method is less effective such as attempting to quit without any support. Attempting to quit without any support is the most commonly cited way to quit smoking in England and as such our category of ‘other methods’ is dominated by this less effective approach (in our sample of quit attempters, ~43% used no support vs 5% who used any aid that is not a vape or prescription/specialist support). The full list of options is indicated in table 1 and in the online supplemental material.

**Table 1:** Categorisation of quit aid use

Category of quit aid	What was used in the most recent quit attempt
Vape	Electronic cigarette or vaping device
Prescription or specialist support	Zyban (bupropion) Champix (varenicline) Cytisine (eg, Tabex, Tactizen or Desmoxan) Nicotine replacement product on prescription or given to you by a health professional Attended one or more Stop Smoking one-to-one counselling/advice/support session(s) Attended a Stop Smoking group
Other method	Nicotine replacement product (eg, patches/gum/inhaler) without a prescription) Tobacco-free nicotine pouch/pod or 'white pouches’ that you place on your gum (eg, Zyn, On!, Nordic Spirit, Velo, Lyft, Skruf), Phoned a smoking helpline A book or booklet Visited www.nhs.uk/smokefree website Visited a website other than Smokefree Used an application (‘app’) on a handheld computer (smartphone, tablet, personal digital assistant) Hypnotherapy Acupuncture Heat-not-burn cigarette (eg, iQOS with HEETS, heatsticks) Juul Allen Carr Easyway session Allen Carr Easyway book The SmokeFree Formula book Other book or booklet Other Willpower alone or nothing

### Disposable vape use

Participants who were currently vaping at the time of the survey were asked which type of device they mainly use (see online supplemental material for full question and responses). Those who reported using a ‘disposable e-cigarette or vaping device (non-rechargeable)’ device were classified as currently using a disposable vape.

### Covariates

#### Time to first cigarette

Cigarette dependence was measured using the time to first cigarette after waking item from the Fagerström Test for Cigarette Dependence, a validated single item measure of nicotine dependence^19^ (≤30 min and >30 min). This is measured for people who currently smoke and, retrospectively, for people who quit smoking (including cigarettes and other forms of combusted tobacco) in the past year.

### Sociodemographic characteristics

Age was modelled as a continuous variable using restricted cubic splines in regression analyses, and as a categorical variable for descriptive results tables (groups: 18–24, 25–34, 35–44, 45–54, 55–64, ≥65 years).

Gender was self-reported as man or woman. Participants also had the option to describe their gender in another way; those who identified in another way were excluded from analyses by gender due to low case numbers (indicated in the footnotes of table 2).

**Table 2:** Results for segmented log-binomial regression models examining the association of the rise in popularity of disposable in England with changes in the annual trend in using a vape, prescription or specialist support and other methods in a quit attempt, respectively

	Vape				Prescription or specialist support				Other methods			
	**RR**	**95% CI**	**F**	**edf**	**RR**	**95% CI**	**F**	**edf**	**RR**	**95% CI**	**F**	**edf**
Pre-disposables trend	0.97	0.93 to 1.01	–	–	0.88	0.80 to 0.97	–	–	1.04	1.01 to 1.06	–	–
Change in trend	1.20	1.12 to 1.29	–	–	0.94	0.79 to 1.11	–	–	0.91	0.88 to 0.95	–	–
Post-disposables trend	1.17	1.04 to 1.31	–	–	0.83	0.64 to 1.08	–	–	0.94	0.89 to 1.01	–	–
Age*	–	–	20.67	1.97	–	–	69.57	1.98			16.40	1.99
Change in trend×age	–	–	1.40	1.32	–	–	0.15	1.00	–	–	6.71	1.40
Change in trend×gender	1.12	1.04 to 1.21	–	–	0.91	0.74 to 1.12	–	–	0.95	0.90-0.99	–	–
Change in trend×social grade	0.99	0.91 to 1.07	–	–	0.97	0.79 to 1.12	–	–	1.02	0.97 to 1.06	–	–
Change in trend×TTFC	0.96	0.88 to 1.03	–	–	0.81	0.66 to 1.00	–	–	1.07	1.02 to 1.12	–	–

Results from separate segmented generalised additive models (log link). All models adjusted for seasonality using a smoothing term with cyclic cubic splines, and the onset of the COVID-19 pandemic (coded 0 to February 2020 and 1 from March 2020). Coefficients multiplied by 12 to report annual trends. Other methods=those who did not use a vape or use prescription/specialist support offered by SSS in their smoking quit attempt.

* Age modelled non-linearly using restricted cubic splines with three knots (placed at the 0th, 50th and 95th percentiles of the data). edf (estimated df) reflects the complexity of a smooth term, with values >1 indicating non-linearity. The F statistic tests whether the term significantly improves model fit, with higher values indicating a significant, non-linear association with the outcome.

edf, effective degrees of freedom; RR, relative risk; SSS, stop smoking services; TTFC, time to first cigarette.

Occupational social grade was categorised as ABC1 (managerial, professional and upper supervisory occupations) and C2DE (manual routine, semiroutine, lower supervisory and long-term unemployed).

### Analysis

The analytic sample consisted of those who smoked and tried to quit smoking in the past year. Two outcomes were examined: (1) making their most recent quit attempt using a vape and (2) making their most recent quit attempt using other methods.

### Segmented regression analysis

A segmented logistic regression analysis assessed the association between the rise in the use of disposable vape use with changes in the trend in the use of vapes in a smoking quit attempt. In an unplanned addition to the preregistered analysis, segmented log-binomial regression models were run, allowing us to report risk ratios and the corresponding relative percentage change in prevalence each year. Further, separate models were run to assess the association between the rise in the use of disposables with changes in the trend in the use of prescription or specialist support and other methods, respectively.

To represent the underlying trend in the pre-disposables period, time was coded sequentially from 1 to n (with n representing the number of waves from July 2016 to December 2023). To account for the change in the trend from the pre- to the post-disposable period, a separate time variable was coded from 1 to m (with m representing the number of waves from June 2021 onward). We assumed that the log-odds of using vapes (or other methods and prescription or specialist support) follow a linear trend within each segment. Coefficients were multiplied by 12 to convert monthly effects into annual trends and risk ratios are reported for the underlying pre-disposable trend and the change in trend from pre-disposables to post-disposables. We used predicted estimates from the models to plot trends in the outcomes in each period.

To account for potential confounding, the above models adjusted for seasonality and effects of COVID-19. Seasonality (month of the year, 0 to 12) was modelled using cyclic cubic splines, ensuring smooth transitions between the beginning and end of each 12-month period.^20^ A binary indicator captured the pandemic’s impact—coded as 0 for data collected up to February 2020 and 1 from April 2020 onward (noting that no data were collected in March 2020)—to adjust for the transition from face-to-face to telephone interviews. Equivalent models were run for the outcome of attempting to quit smoking using prescription or specialist support and other methods.

#### Interaction models

Further separate segmented regression models included an interaction between the change in trend and key sociodemographic variables (age, gender and social grade, respectively), and cigarette dependence (time to first cigarette) to assess whether time trends in these outcomes differed according to these indicators. Age was modelled using restricted cubic splines with three knots (placed at the 0th, 50th and 95th percentiles of the data), to allow for a non-linear relationship between age and use of a quit aid.

### Sensitivity analyses

#### Shifting the timing of the interruption forwards

To assess whether the chosen month selected for the interruption in the primary segmented regression models affected the results, we conducted sensitivity analyses shifting the interruption point by 1 and 2 months later (ie, July and August 2021). This addressed uncertainty around the exact month when disposable vape use began rising, and tested whether our choice of interruption point, which was necessarily somewhat arbitrary, influenced the findings.

#### Modelling the changes in outcomes associated with the prevalence of disposable vaping as a continuous variable

The primary segmented regression uses the interruption as a proxy for the start of the rise in the use of disposable vapes in a quit attempt but does not directly examine the association between disposable vaping prevalence and overall vape use in a quit attempt. To triangulate with the primary analysis, we modelled whether the changes in the prevalence of vape use in a quit attempt were associated with the prevalence of disposable vaping as a continuous variable rather than an abrupt interruption in June 2021. This allowed us to examine whether a percentage-point increase in disposable vaping prevalence was associated with increases in the use of vapes in a smoking quit attempt. In these analyses, we computed aggregate weighted prevalence estimates for each combination of month and age group (18–24, 25–44, ≥45 years) and used linear regression to estimate the association between disposable vaping prevalence and the prevalence of vape use in a smoking quit attempt in the subsequent month (ie, one-period lag), after adjustment for underlying trends (assumed to be linear), seasonality (using cyclic splines), age group and the change in interview modality. Durbin-Watson tests were used to check for autocorrelation. To account for potential non-independence of observations within months, we computed cluster-robust standard errors using the sandwich package in R, specifying clustering at the month level.

As an unplanned addition to the preregistered sensitivity analysis, we ran equivalent models with the outcomes of the use of prescription or specialist support and other quit methods, respectively, to correspond fully to the results reported in the primary segmented regression analyses.

#### Disposable vape use as a proportion of overall vape use

Finally, to provide further information on the extent to which the rise in the use of vapes to support smoking quit attempts may be due to the use of disposable devices specifically, we also computed the annual weighted prevalence of disposable use among people who used a vape in their past year smoking quit attempt and were still vaping (data were not collected on the device type specifically used in the quit attempt).

Data were analysed in R V.4.4.1 using the tidyverse,^21^ mgvc^22^ and survey^23^ packages.

### Missing data

Multiple imputation would have been considered if missingness was above 5%, but because it was low (0.1%) the analyses used data from complete cases.

### Role of the funding source

The STS funders were not involved in the study design or conduct; collection, management, analysis or interpretation of data; preparation, review or approval of the manuscript; or the decision to submit the manuscript for publication. All authors were responsible for the decision to submit the manuscript.

### Patient and public involvement

The wider STS is discussed several times a year with a diverse patient and public involvement group, and the authors regularly attend and present at meetings at which patients and members of the public are included. Interaction and discussion at these events help shape the broad research priorities and questions. A mechanism also exists for generalised input from the wider public: each month, interviewers seek feedback on the questions from all respondents, who are representative of the English population. This feedback is limited and usually relates to understanding of questions and item options. No patients or members of the public were involved in setting the research questions or the outcome measures, nor were they involved in the design and implementation of this specific study.

## Results

A total of 145 787 (unweighted) adults aged ≥18 years in England were surveyed between July 2016 and December 2023. Of these, 16 464 individuals smoked but did not try to quit in the past year, and 8323 adults had smoked and tried to quit in the past year. Among people who smoked but did not try to quit in the past 12 months, the prevalence of currently using a vape (for any reason) was 17.2% (95% CI 16.6% to 17.9%). In contrast, among those who had tried to quit smoking in the past 12 months, use of a vape in their most recent attempt was 31.1% (95% CI 30.0% to 32.2%). Our analytic sample consisted of all those who tried to quit smoking in the past 12 months (47.5% women; mean (SD) age: 39.2 (15.5); other characteristics of the sample are presented in online supplemental table S1)).

**Figure 1:**
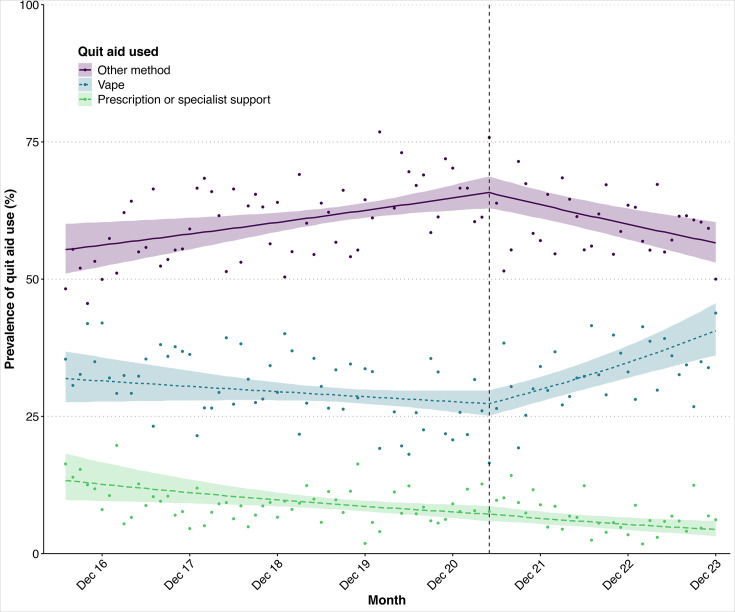
Trends in the use of a vape and less effective methods, in an attempt to stop smoking pre and post the growth in disposable vape use, July 2016 to December 2023. Lines represent modelled weighted prevalence by month from segmented generalised additive models (log link). Shaded bands represent 95% CIs. Points represent unmodelled weighted prevalence data by month. The vertical dashed line indicates the start of the rise in popularity of vaping using disposable devices in June 2021. Other methods=attempts that did not use a vape, or prescription/specialist support.

Results from the primary segmented regression analyses are shown in figure 1 and table 2 (logistic regression results in online supplemental table S2). During the pre-disposable vapes period (before June 2021), the prevalence of the use of vapes in a smoking quit attempt underwent an uncertain relative decline of 3.1% per year. However, this trend reversed when disposable vapes became popular with a relative year-on-year increase in prevalence of 16.5% per year from June 2021 onwards (from 27.7% in June 2021 to 40.6% in December 2023). In contrast, the prevalence of people attempting to quit smoking using other methods was increasing by 3.6% per year before June 2021, after which the trend reversed and there was a relative year-on-year uncertain decline in prevalence of 5.6% (from 65.4% in June 2021 to 56.6% in December 2023). The use of prescription or specialist support underwent a relative decline by 12.0% per year in the pre-disposables period, and there was no apparent change in trend from June 2021 onward. Sensitivity analyses shifting the interruption time point later by one or 2 months (online supplemental table S3) produced similar results, showing that the findings were not sensitive to the specific month chosen as the starting point for increased use of disposables.

Trends in vape use in a quit attempt and in the use of other methods differed by gender. Women experienced steeper growth in vaping compared with men (women: 26.7% to 45.5%; men: 28.6% to 36.4%) and greater declines in the use of other methods (women: 65.9% to 52.4%; men: 65.5% to 60.2%) between June 2021 and December 2023 (table 1 and online supplemental figure S1). In terms of age, the reversal in the trend of vape use in a quit attempt was observed across all age groups. However, the pattern of age-related differences remained stable, with younger adults consistently reporting higher vape use (table 1 and online supplemental figure S2). That is, while vape use in a quit attempt increased across all age groups in the post-disposables period, the relative rate of increase was proportionate across ages. In contrast, the age-related pattern in the use of other methods changed in the post-disposables period. Although all age groups showed similar rates of increase before June 2021, the trend stalled—but did not reverse—among the oldest groups after disposables became popular (table 1 and online supplemental figure S2). Trends in the use of other methods in a quit attempt also differed according to time to first cigarette after waking, with a steeper decline from June 2021 to December 2023 for those who smoked their first cigarette at least 30 min after waking (from 71.3% to 58.0%), compared with those who smoked within 30 min of waking (56.7% to 54.4%) (table 1 and online supplemental figure S3). In contrast, the declining trend in the use of prescription or specialist support may have accelerated slightly from June 2021 in the ≤30 min time to first cigarette group (10.7% to 5.0%) relative to the >30 min group (4.8% to 3.8%), with low use apparent across both groups by the end of the period. Trends did not appear to change according to social grade between the pre-disposables and post-disposables periods for all quit aids examined (table 1 and online supplemental figure S4).

Results were comparable when we examined changes in the use of vapes in a quit attempt associated with the prevalence of disposable vaping (adjusted for age, seasonality and change in survey modality) as a continuous variable rather than an abrupt interruption. For each 1 percentage-point increase in disposable vaping prevalence, the prevalence of vaping in a quit attempt (in the subsequent month) rose by an estimated 0.66 percentage points (95% CI 0.04 to 1.26), while the use of other methods declined by 0.82 percentage points (−1.40 to −0.24) (online supplemental table S4). The use of prescription or specialist support rose by 0.16 percentage points (−0.02 to 0.34), but the lower CI indicates that no change, or even a slight reduction, cannot be ruled out.

**Figure 2:**
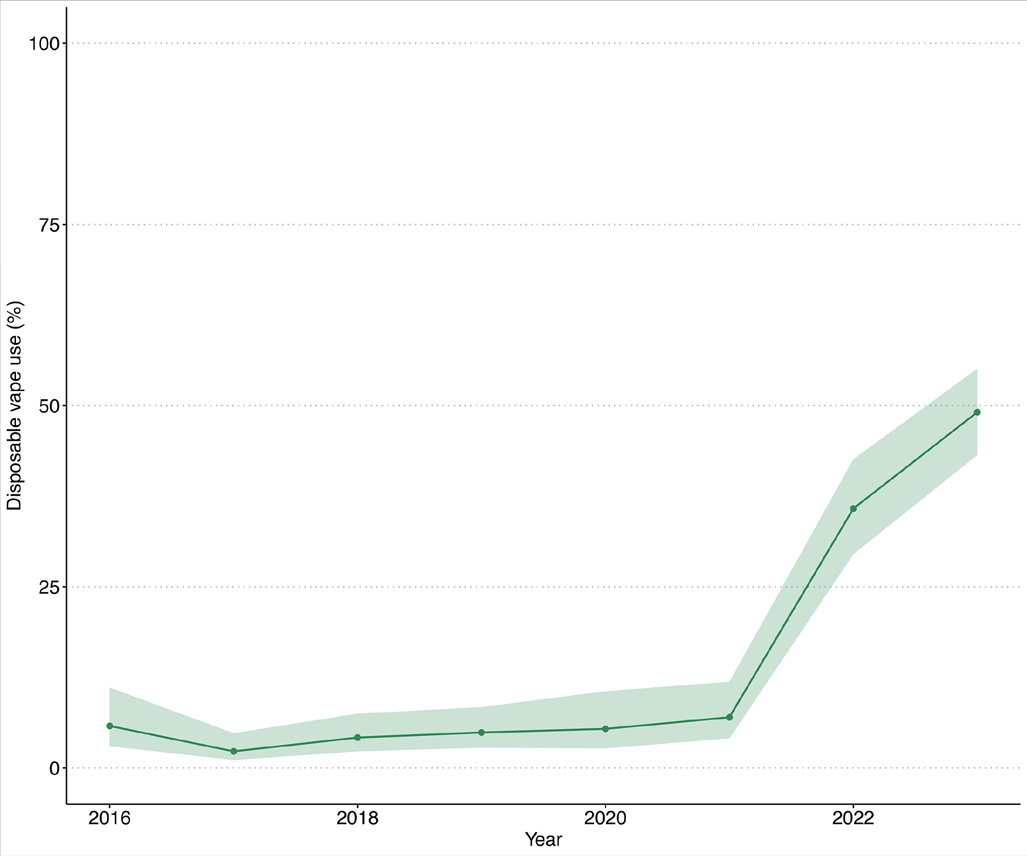
The annual weighted prevalence of disposable vape use among people who used a vape in their past-year smoking quit attempt and were still vaping. Shaded band represents 95% CIs around the weighted percentage for disposable vape use in each year.

The annual weighted prevalence of disposable vape use among people who used a vape in their past-year smoking quit attempt and were still vaping at the time of the survey (irrespective of their smoking status) increased from approximately 5% each year up to 2020, to 49.1% in 2023 (figure 2 and online supplemental table S5). This is supportive of the observed increase in the use of vapes in a quit attempt being driven by the widespread availability of disposable vapes to the market in June 2021.

## Discussion

The rise in popularity of disposable vapes in England was associated with a reversal of the previously declining trend in the use of vapes among people attempting to quit smoking and a decrease in the use of other (non-prescription or specialist support) methods of quitting. From June 2021, the proportion of quit attempts supported by a vape increased by 16.5% per year (from 27.7% in June 2021 to 40.6% in December 2023). Concomitantly, there was a decline in the use of other (non-prescription or specialist support) methods for quit attempts of 5.6% per year (from 65.4% to 56.6%). Recent research in England has shown that whereas attempts to quit smoking unaided have one-third lower odds of being successful compared with attempts using some kind of support, vape-supported quit attempts are approximately twice as likely to be successful than those that do not involve the use of a vape.^11^ This is supported by evidence from RCTs^24^ and observational studies.^25^

Results from two sets of sensitivity analyses correspond to the primary segmented regression analysis. First, we found that each percentage-point increase in disposable vaping prevalence was associated with a 0.66 percentage-point increase in the use of vapes in a smoking quit attempt. While this does not eliminate the possibility that unmeasured factors contributed to the changes, there were no clear population-level policies or a meaningful rise in the use of other quit aids that occurred around the same time that novel disposable vapes entered the market.^11^ Second, the annual weighted prevalence of disposable use among people who used a vape in their recent attempt to quit smoking and were still vaping at the time of the survey increased from approximately 1 in 20 past-year smokers across the pre-disposables period (2016–2021) to one in two by 2023. When considered alongside the declining trend in vape use in a quit attempt up to May 2021 and the subsequent rise thereafter, these data are supportive of the observed increase in the use of vapes in a quit attempt being driven by the entry of disposable vapes to the market in June 2021.

These results are important to consider in light of the ban on the sale of disposable vapes in England coming into effect in June 2025.^17^ Following the announcement of the disposables ban in January 2024, increases in vaping prevalence stalled, and people increasingly switched from using disposable vapes to devices that can be refilled and recharged.^26^ Many of these devices are like disposables in their design, nicotine content, branding, price and availability.^27^ If these product characteristics drive popularity, such substitution is likely to limit the impact of the ban on vaping in smoking quit attempts or vaping in young people (unless coverage of the ban notably affects perceptions of vapes in general). To inform future regulation, further research should examine device use in the period following the implementation of the disposables ban and understand whether certain product characteristics are important drivers of their use in smoking quit attempts. To reduce the access to and appeal of disposable-like reusable devices to young people while minimising reductions in their use for adult smoking cessation, future targeted regulation could include restrictions on device packaging and marketing, point-of-sale displays and taxation.^4^ One unintended consequence of regulation could be rises in cigarette smoking in youth,^28^ but in the context of the impending smoke-free generation policy (where those born after 2008 will never be able to legally be sold tobacco products)^16^ this concern should diminish over time assuming effective enforcement.

When examining changes in quit attempts according to sociodemographic characteristics, results from our analyses indicate that the decreases in quit attempts using other (non-prescription or specialist support) methods from June 2021 were more pronounced among younger relative to older adults, women relative to men, and lower dependence relative to higher dependence adults. If the concomitant increases in quit attempts using vapes in these groups translated into greater quit success and long-term smoking cessation, then this could have contributed to differential declines in smoking.^3^ Our results also indicate that the secular decline in the use of prescription and specialist support may have accelerated in the postdisposables period among more dependent individuals. However, given that this group is more likely to seek specialist support to quit than less dependent individuals, this accelerated decline from June 2021 is likely influenced by the major disruption to the supply of varenicline in the UK that occurred around the same time as the rise in disposable vapes.^13^ While it is possible that disposable vapes contributed to people switching away from the use of prescription and specialist support, this possibility cannot be disentangled from the reverse effect: that the varenicline disruption led to people seeking out alternative methods to quit, such as vapes.

This study has several limitations. First, our measure of aids used in a quit attempt refers to the most recent quit attempt in the past year, which may miss nuances among individuals who made multiple serious attempts to stop smoking during that period. Second, we used an observational cross-sectional time-series design, meaning changes cannot conclusively be attributed to increases in disposable vape use. However, triangulation through primary and sensitivity analyses strengthens the plausibility of the associations we identified. Future research using controlled interrupted time series designs could be valuable for evaluating the impact of policy interventions such as the disposable vape ban on the use of e-cigarettes in smoking quit attempts. For instance, comparing the effect in young people (a group in which disposable vapes are popular) with that in older adults (where disposable use is rare) with the expectation that older groups’ use of e-cigarettes in quit attempts should be largely unaffected by a disposable ban.

In conclusion, the rise in popularity of disposable vapes in England from mid-2021 was associated with an increase in the use of vapes during smoking quit attempts and a reduction in use of other less effective (non-prescription or specialist support) methods of quitting. After disposable vapes are banned in England, future regulation of similar disposable-like reusable vapes will need to consider the potential benefits of the devices towards smoking cessation while designing regulation to restrict access to youth.

## Supplementary material

10.1136/bmjph-2025-004422 online supplemental file 1

## Data Availability

Data are available on reasonable request.
