# Supplementary material for: Changes in the use of e-cigarettes to stop smoking among adults following the rise of disposable vapes: a repeat cross-sectional survey 2016–2023 in England
Source: BMJ Public Health. 2026 Jun 28;4(2):e004422. doi: 10.1136/bmjph-2025-004422 (PMC13343014; doi:10.1136/bmjph-2025-004422)
Supplement: online supplemental file 1 [file bmjph-4-2-s001.docx]

**SUPPLEMENTARY MATERIAL**

**Measures**

*Smoking status*

Assessed by asking participants which of the following best applies to them:

1. ‘I smoke cigarettes (including hand-rolled) every day’
2. ‘I smoke cigarettes (including hand-rolled), but not every day’
3. ‘I do not smoke cigarettes at all, but I do smoke tobacco of some kind (e.g. pipe, cigar or shisha)’
4. ‘I have stopped smoking completely in the last year’
5. ‘I stopped smoking completely more than a year ago’
6. ‘I have never been a smoker (i.e. smoked for a year or more)’

Those who respond a-d are considered past-year smokers.

*Use of an e-cigarette as a smoking quit aid*

People who smoked in the past year and reported a past-year quit attempt were asked to indicate which, if any, of the following they used try to stop smoking during their most recent serious quit attempt:

1. Nicotine replacement product (e.g. patches\gum\inhaler) without a prescription
2. Nicotine replacement product on prescription or given to you by a health professional
3. Zyban (bupropion)
4. Champix (varenicline)
5. Cytisine (e.g. Tabex, Tactizen or Desmoxan)
6. Tobacco-free nicotine pouch/pod or 'white pouches' that you place on your gum (e.g., Zyn, On!, Nordic Spirit, Velo, Lyft, Skruf)
7. Attended a Stop Smoking group
8. Attended one or more Stop Smoking one-to-one counselling\advice\support session\s
9. Phoned a Smoking Helpline
10. A book or booklet
11. Visited www.nhs.uk\smokefree website
12. Visited a website other than Smokefree
13. Used an application ('app') on a handheld computer (smartphone, tablet, PDA)
14. Hypnotherapy
15. Acupuncture
16. Electronic cigarette or vaping device
17. Heat-not-burn cigarette (e.g. iQOS with HEETS, heatsticks)
18. Juul
19. Allen Carr Easyway session
20. Allen Carr Easyway book
21. The SmokeFree Formula book
22. Other book or booklet
23. Other

Those who indicated they used an electronic cigarette or vaping device were classified as using a vape in their most recent smoking quit attempt. Those who reported using a prescribed nicotine replacement product or medication (bupropion, varenicline, or cytisine), or a form of specialist behavioural smoking cessation support (a stop smoking group, or one-to-one counselling) were classified as using standard evidence-based support typically available through NHS specialist Stop Smoking Services (SSS) or primary care. All others (i.e. those who did not use a vape or any of these prescription or specialist support methods) were classified as attempting to quit using other, less effective methods.".

*Vaping device*

Those who report currently using a vape were asked which type of device they mainly use:

1. A disposable e-cigarette or vaping device (non-rechargeable)
2. An e-cigarette or vaping device that uses replaceable pre-filled cartridges (rechargeable)
3. An e-cigarette or vaping device with a tank that you refill with liquids (rechargeable)
4. A modular system that you refill with liquids (you use your own combination of separate devices batteries, atomizers etc.)
5. Don't know

Those who use a “disposable e-cigarette or vaping device (non-rechargeable)” device (a) will be classified as currently using a disposable vape.

**Table S1: Weighted sample characteristics.**

|  | **Overall** | **Pre-disposables** | **Post-disposables** |
| --- | --- | --- | --- |
| **Characteristic** | N = 8,907 | N = 5,613 | N = 3,294 |
| **Mean age (SD)** | 39.2 (15.5) | 39.7 (15.5) | 38.4 (15.3) |
| **Age** |  |  |  |
| 18-24 | 1,741 (19.6%) | 1,030 (18.3%) | 712 (21.6%) |
| 25-44 | 4,160 (46.7%) | 2,620 (46.7%) | 1,539 (46.7%) |
| 45-54 | 1,355 (15.2%) | 907 (16.2%) | 448 (13.6%) |
| 55-64 | 944 (10.6%) | 587 (10.5%) | 357 (10.8%) |
| 65+ | 707 (7.9%) | 470 (8.4%) | 237 (7.2%) |
| **Gender^a^** |  |  |  |
| Women | 4,235 (47.5%) | 2,668 (47.5%) | 1,567 (47.6%) |
| Men | 4,672 (52.5%) | 2,945 (52.5%) | 1,727 (52.4%) |
| **Social grade** |  |  |  |
| ABC1 | 3,769 (42.3%) | 2,360 (42.0%) | 1,409 (42.8%) |
| C2DE | 5,138 (57.7%) | 3,253 (58.0%) | 1,885 (57.2%) |
| **Vape use in quit attempt** | 2,767 (31.1%) | 1,684 (30.0%) | 1,084 (32.9%) |
| **Other (non-prescription or specialist support in quit attempt)** | 5,397 (60.6%) | 3,415 (60.8%) | 1,983 (60.2%) |
| **Prescription or specialist support in quit attempt** | 742 (8.3%) | 515 (9.2%) | 227 (6.9%) |
| **Time to first cigarette^b^** |  |  |  |
| ≤30 minutes | 5,105 (58.9%) | 3,245 (57.8%) | 1,860 (56.5%) |
| >30 minutes | 3,561 (41.1%) | 2,286 (40.7%) | 1,275 (38.7%) |
| Missing | 240 (2.7%) | 82 (1.5%) | 158 (4.8%) |

Categorical variables shown as n (%).
Pre-disposables period was from July 2016 to May 2021. Post-disposables period was from June 2021 to December 2023.
^a^Under gender, 0.7% of participants identified “in another way”.
^b^Time to first cigarette is measured retrospectively for all those who quit smoking (including cigarettes and other forms of combusted tobacco) in the past year.

**Table S2: Results for segmented logistic regression models examining the association of the rise in popularity of disposable in England with changes in the annual trend in using a vape in a quit attempt, and no prescription or specialist support in a quit attempt, respectively.**

|  | **Vape** | | | | **Prescription or specialist support** | | | | **Other methods** | | | |
| --- | --- | --- | --- | --- | --- | --- | --- | --- | --- | --- | --- | --- |
|  | **OR** | **95% CI** | **F** | **edf** | **OR** | **95% CI** | **F** | **edf** | **OR** | **95% CI** | **F** | **edf** |
| Pre-disposables trend | 0.96 | 0.90-1.02 | - | - | 0.88 | 0.79-0.97 | - | - | 1.10 | 1.03-1.16 | - | - |
| Change in trend | 1.31 | 1.18-1.46 | - | - | 0.93 | 0.78-1.12 | - | - | 0.79 | 0.71-0.87 | - | - |
| Post-disposables trend | 1.25 | 1.06-1.49 | - | - | 0.83 | 0.64-1.07 | - | - | 0.86 | 0.73-1.01 | - | - |
| Change in trend X Age* | - | - | 1.72 | 1.40 | - | - | 0.27 | 1.00 | - | - | 6.65 | 1.37 |
| Change in trend X Gender | 1.20 | 1.07-1.37 | - | - | 0.89 | 0.71-1.12 | - | - | 0.88 | 0.79-0.99 | - | - |
| Change in trend X Social grade | 0.98 | 0.87-1.10 | - | - | 0.97 | 0.77-1.22 | - | - | 1.12 | 0.93-1.17 | - | - |
| Change in trend X TTFC | 0.94 | 0.83-1.06 | - | - | 0.79 | 0.63-1.00 | - | - | 1.18 | 1.05-1.32 | - | - |

Results from separate segmented generalized additive models (logit link). All models adjusted for seasonality using a smoothing term with cyclic cubic splines, and the onset of the covid-19 pandemic (coded 0 to February 2020 and 1 from March 2020). Coefficients multiplied by 12 to report annual trends.
OR=Odds Ratio.
edf=effective degrees of freedom
*Age modelled non-linearly using restricted cubic splines with three knots (placed at the 0th, 50th, and 95th percentiles of the data).

**Table S3: Results for segmented log-binomial and logistic regression models examining the association of the rise in popularity of disposable in England with changes in the annual trend in using a vape in a quit attempt, and no prescription or specialist support in a quit attempt, respectively. Timing of the interruption shifted later by one and two months.**

|  | **Vape** | | **Prescription or specialist support** | | **Other method** | |
| --- | --- | --- | --- | --- | --- | --- |
| **Log-binomial regression** | **RR** | **95% CI** | **RR** | **95% CI** | **RR** | **95% CI** |
| Interruption shifted +1 month |  |  |  |  |  |  |
| Pre-disposables trend | 0.97 | 0.93-1.01 | 0.88 | 0.80-0.97 | 1.03 | 1.01-1.06 |
| Change in trend | 1.20 | 1.12-1.30 | 0.93 | 0.78-1.11 | 0.91 | 0.88-0.95 |
| Post-disposables trend | 1.17 | 1.04-1.31 | 0.83 | 0.64-1.07 | 0.94 | 0.88-1.01 |
| Interruption shifted +2 months |  |  |  |  |  |  |
| Pre-disposables trend | 0.97 | 0.93-1.02 | 0.88 | 0.81-0.97 | 1.03 | 1.01-1.06 |
| Change in trend | 1.20 | 1.12-1.30 | 0.93 | 0.77-1.11 | 0.91 | 0.87-0.95 |
| Post-disposables trend | 1.17 | 1.04-1.32 | 0.83 | 0.64-1.07 | 0.94 | 0.88-1.01 |
| **Logistic regression** | **OR** | **95% CI** | **OR** | **95% CI** | **OR** | **95% CI** |
| Interruption shifted +1 month |  |  |  |  |  |  |
| Pre-disposables trend | 0.96 | 0.90-1.03 | 0.88 | 0.79-0.97 | 1.09 | 1.03-1.16 |
| Change in trend | 1.31 | 1.18-1.47 | 0.93 | 0.77-1.12 | 0.79 | 0.71-0.87 |
| Post-disposables trend | 1.26 | 1.06-1.50 | 0.83 | 0.64-1.07 | 0.86 | 0.73-1.01 |
| Interruption shifted +2 months |  |  |  |  |  |  |
| Pre-disposables trend | 0.96 | 0.90-1.02 | 0.88 | 0.79-0.97 | 1.09 | 1.02-1.15 |
| Change in trend | 1.31 | 1.18-1.46 | 0.92 | 0.76-1.12 | 0.79 | 0.71-0.88 |
| Post-disposables trend | 1.27 | 1.06-1.51 | 0.83 | 0.74-1.07 | 0.86 | 0.73-1.01 |

**Table S4**: Linear regression estimating the association between monthly prevalence of disposable e-cigarette use as a continuous variable, and the prevalence of vape use in a smoking quit attempt, use of prescription or specialist support, and other less effective quit aid use, in the subsequent month (i.e., a one-period lag)

|  | **Regression coefficient** | |
| --- | --- | --- |
|  | Beta^a^ | 95% CI^b^ |
| **Vape** | +0.66% | +0.05% to +1.26% |
| **Prescription or specialist support** | +0.16% | -0.02% to +0.34% |
| **Other method** | -0.82% | -1.40% to -0.24% |

Linear regression models were all run using aggregate monthly data, with the outcome of use of a vape in the most recent serious quit attempt, prescription or specialist support, and use of other less effective methods, respectively. Predictors were the prevalence of disposable e-cigarette vaping in the previous month, age, seasonality (modelled using splines), temporal trend, and the change in modality from face-to-face to telephone interview after March 2020.
^a^Beta represents the percentage point change in outcomes for every one percentage point increase in the prevalence of disposable e-cigarette vaping. Results from Durbin-Watson tests (vape use p-value = 0.48; prescription or specialist support p-value = 0.78; other less effective method p-value = 0.98) indicating little evidence for autocorrelation.
^b^95% CIs computed using cluster-robust standard errors, specifying clustering at the month level.

**Table S5: The annual weighted prevalence of disposable vape use among people who used a vape in their past year smoking quit attempt and were still vaping.**

| **Year** | **Disposable %** | **Lower 95% CI** | **Upper 95% CI** | **N** |
| --- | --- | --- | --- | --- |
| 2016 | 5.8 | 3.0 | 11.1 | 164 |
| 2017 | 2.3 | 1.1 | 4.8 | 313 |
| 2018 | 4.2 | 2.3 | 7.5 | 275 |
| 2019 | 4.9 | 2.8 | 8.4 | 236 |
| 2020 | 5.4 | 2.7 | 10.6 | 210 |
| 2021 | 7.0 | 4.1 | 11.9 | 275 |
| 2022 | 35.8 | 29.5 | 42.6 | 260 |
| 2023 | 49.1 | 43.2 | 55.1 | 365 |

**Figure S1:** Trends in the use of a vape, prescription or specialist support, or less effective methods among men and women, respectively, in attempt to stop smoking pre and post the growth in disposable vape use, July 2016 to December 2023.


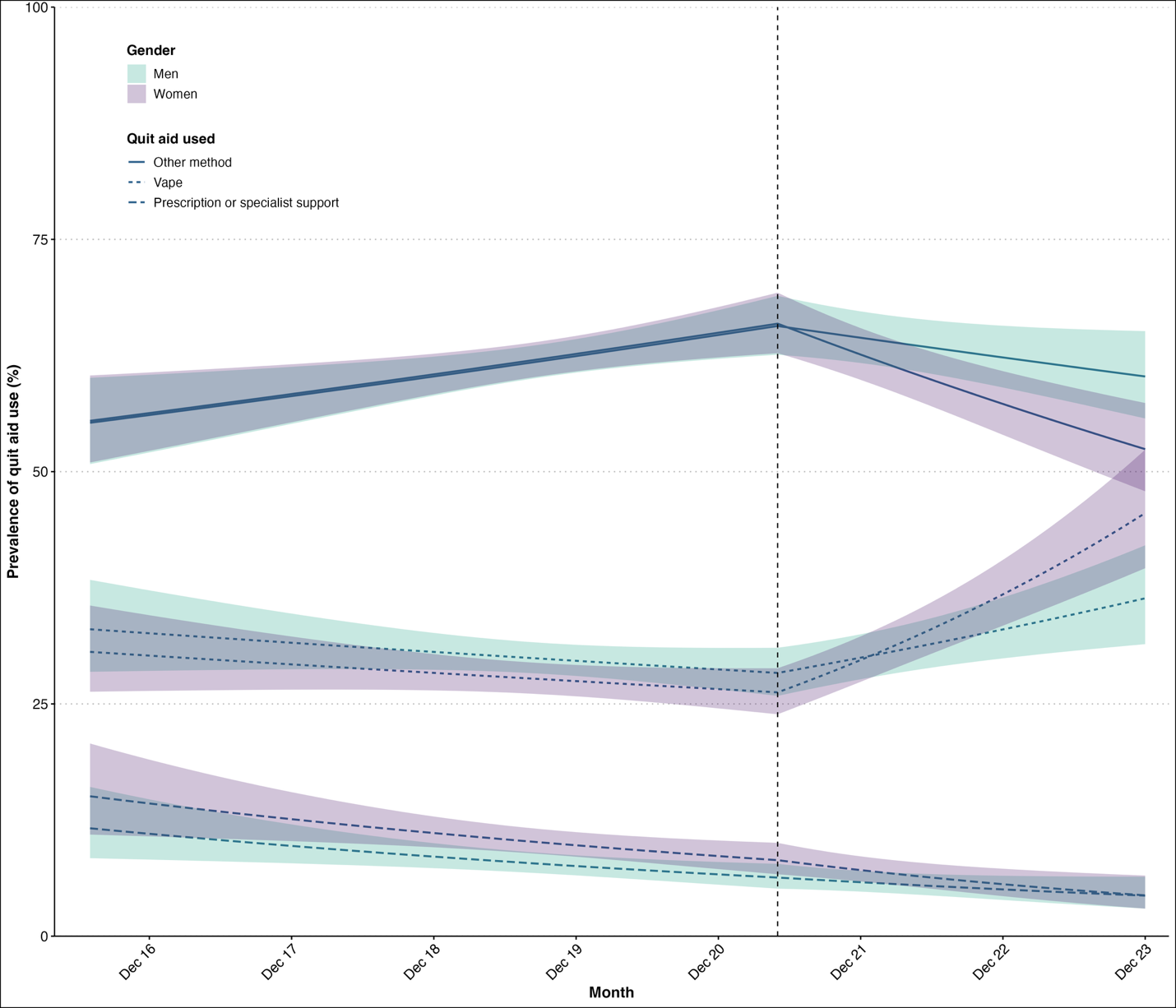


Lines represent modelled weighted prevalence by month from segmented generalized additive models (log link). Shaded bands represent 95% confidence intervals. Points represent unmodelled weighted prevalence data by month. The vertical dashed line indicates the start of the rise in popularity of vaping using disposable devices in June 2021.
Other methods = attempts that did *not* use a vape, or prescription/specialist support offered by SSS).

**Figure S2:** Trends in the use of a vape, prescription or specialist support, or less effective methods by age, in attempt to stop smoking pre and post the growth in disposable vape use, July 2016 to December 2023.


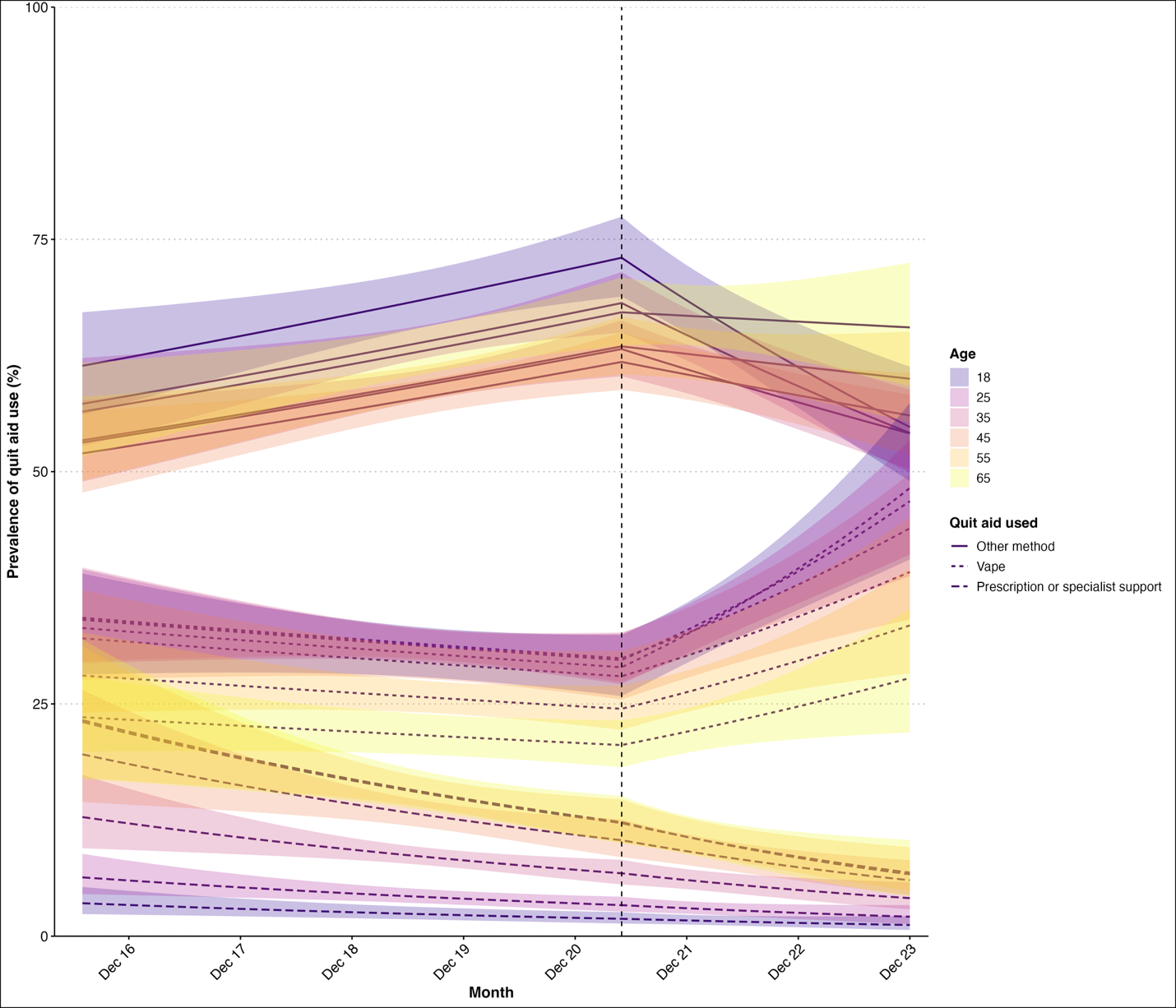


Lines represent modelled weighted prevalence by month from segmented generalized additive models (log link) for six specific years of age (18, 25, 35, 45, 55, and 65 years). Shaded bands represent 95% confidence intervals. The vertical dashed line indicates the start of the rise in popularity of vaping using disposable devices in June 2021.
Age modelled non-linearly using restricted cubic splines with three knots (placed at the 0th, 50th, and 95th percentiles of the data).
Other methods = attempts that did *not* use a vape, or prescription/specialist support offered by SSS).

**Figure S3:** Trends in the use of a vape, prescription or specialist support, or less effective methods by time to first cigarette after waking, in attempt to stop smoking pre and post the growth in disposable vape use, July 2016 to December 2023.


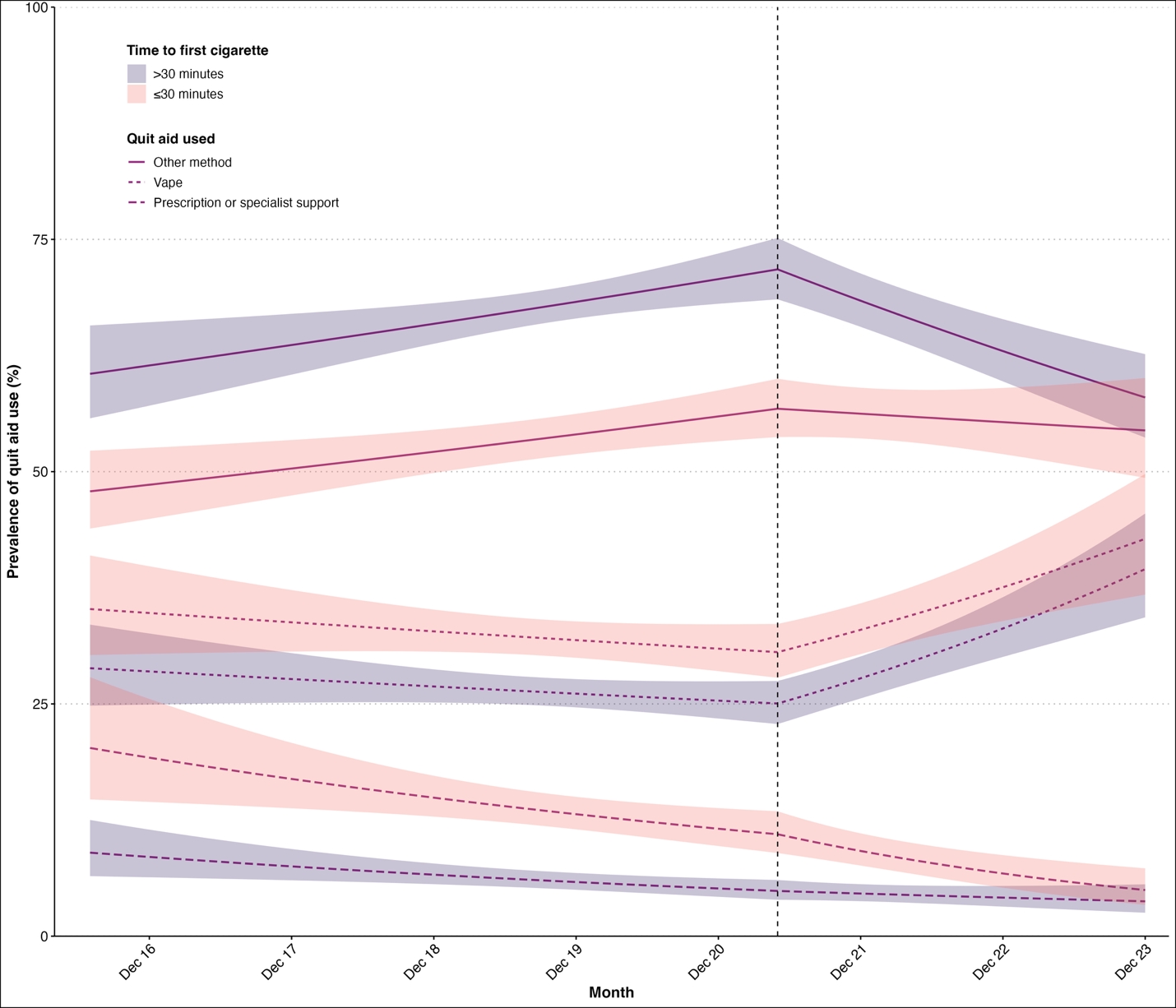


Lines represent modelled weighted prevalence by month from segmented generalized additive models (log link). Shaded bands represent 95% confidence intervals. Points represent unmodelled weighted prevalence data by month. The vertical dashed line indicates the start of the rise in popularity of vaping using disposable devices in June 2021.
Other methods = attempts that did *not* use a vape, or prescription/specialist support offered by SSS).

**Figure S4:** Trends in the use of a vape, prescription or specialist support, or less effective methods by social grade, in attempt to stop smoking pre and post the growth in disposable vape use, July 2016 to December 2023.


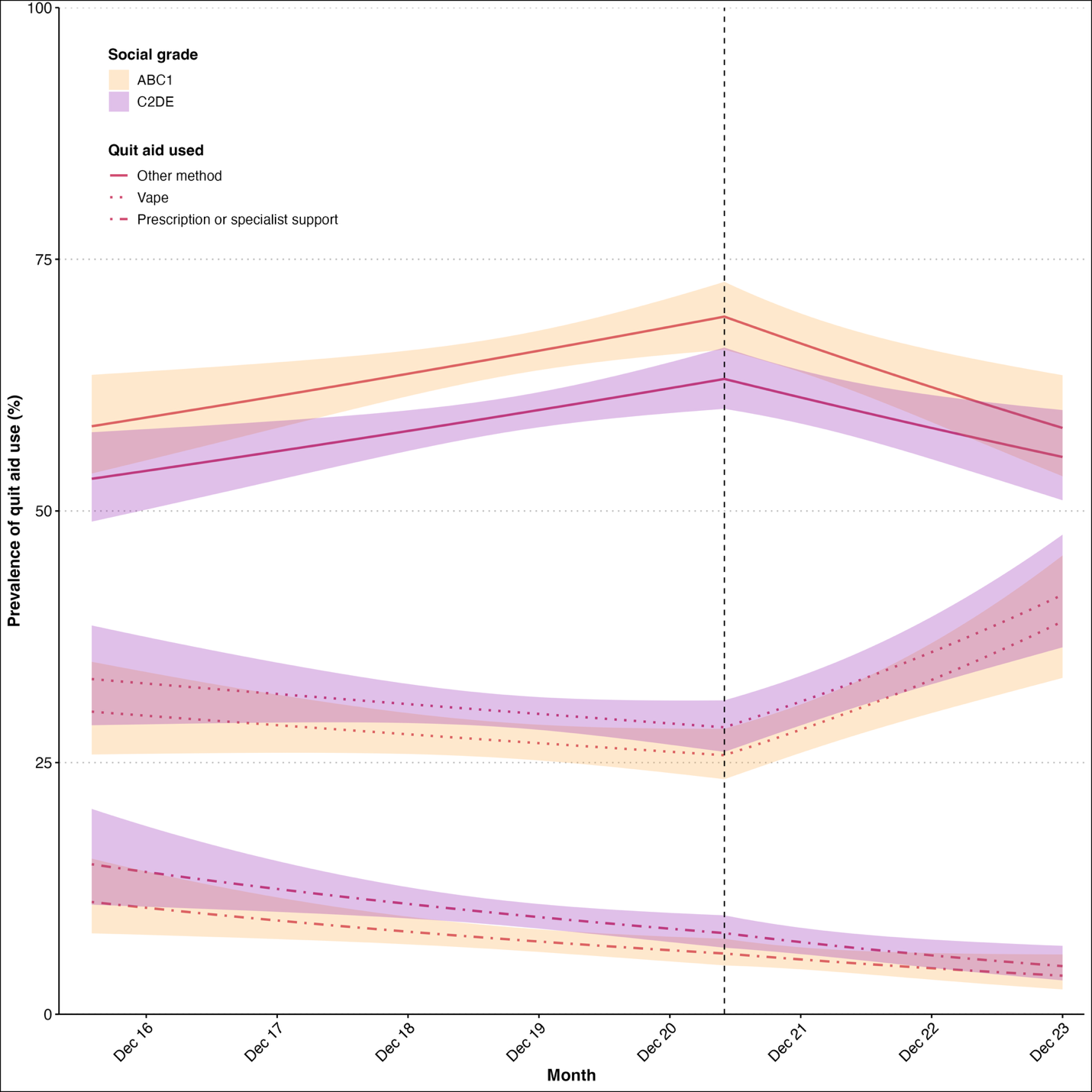


Lines represent modelled weighted prevalence by month from segmented generalized additive models (log link). Shaded bands represent 95% confidence intervals. Points represent unmodelled weighted prevalence data by month. The vertical dashed line indicates the start of the rise in popularity of vaping using disposable devices in June 2021.
Other methods = attempts that did *not* use a vape, or prescription/specialist support offered by SSS).
